# The choice of self-rated health measures matter when predicting mortality: evidence from 10 years follow-up of the Australian longitudinal study of ageing

**DOI:** 10.1186/1471-2318-10-18

**Published:** 2010-04-20

**Authors:** Kerry A Sargent-Cox, Kaarin J Anstey, Mary A Luszcz

**Affiliations:** 1 Ageing Research Unit, Centre for Mental Health Research, Australian National University, Canberra, Australia; 2 School of Psychology and Flinders Centre for Ageing Studies, Flinders University, Adelaide, Australia

## Abstract

**Background:**

Self-rated health (SRH) measures with different wording and reference points are often used as equivalent health indicators in public health surveys estimating health outcomes such as healthy life expectancies and mortality for older adults. Whilst the robust relationship between SRH and mortality is well established, it is not known how comparable different SRH items are in their relationship to mortality over time. We used a dynamic evaluation model to investigate the sensitivity of time-varying SRH measures with different reference points to predict mortality in older adults over time.

**Methods:**

We used seven waves of data from the Australian Longitudinal Study of Ageing (1992 to 2004; N = 1733, 52.6% males). Cox regression analysis was used to evaluate the relationship between three time-varying SRH measures (global, age-comparative and self-comparative reference point) with mortality in older adults (65+ years).

**Results:**

After accounting for other mortality risk factors, poor global SRH ratings increased mortality risk by 2.83 times compared to excellent ratings. In contrast, the mortality relationship with age-comparative and self-comparative SRH was moderated by age, revealing that these comparative SRH measures did not independently predict mortality for adults over 75 years of age in adjusted models.

**Conclusions:**

We found that a global measure of SRH not referenced to age or self is the best predictor of mortality, and is the most reliable measure of self-perceived health for longitudinal research and population health estimates of healthy life expectancy in older adults. Findings emphasize that the SRH measures are not equivalent measures of health status.

## Background

Self-rated health (SRH) is a widely used measure for health status in public health and epidemiological research due to strong associations with other subjective and objective measures of well-being, health outcomes and mortality [e.g. [1]]. The multidimensional concept of health that is encapsulated within a single global SRH response is considered by World Health Organisation (WHO[2]) and the Euro-REVES 2 [3] project to be one of the best indicators of health at the individual and population level. Both of these organisations have extensively investigated the relationship between global SRH and health outcomes and recommended the measure to estimate policy relevant data on aspects of public health such as healthy life expectancy and mortality [4].

The most commonly used SRH measure has a global or current reference point (i.e. how would you rate your health in general/at the present time?). A comparative reference point is also often used to anchor the assessment, such as comparing current health to previous health (self-comparative), or same-aged peers (age-comparative). All these forms of the SRH item are in use in health surveys around the world as an indicator of older adults' years lived in good health [e.g. [3]]. Despite their extensive use, and the robust relationship between poor ratings of SRH and major health outcomes, there is scant research that has compared how these SRH measures perform in older adult populations; in particular it has not been established how the SRH measures compare in their relationship with mortality.

In the few studies that have compared the association between SRH items and mortality, mixed results have been reported. Manderbacka, Kareholt, Martikainen and Lundberg [5] found the predictive quality of global and age-comparative SRH items was dependent on gender, with the age-comparative item being a better predictor of mortality for males than females in simultaneous models. In contrast, Vuorisalmi, Lintonen and Jylhä [6] reported both an age-comparative and global comparative item was a stronger predictor of mortality for males than females. Even less attention has been paid to the self-comparative item, and the temporal reference point that is used in the few studies has been broad (i.e. ranging from 5 to 10 years previous). As the interval over which participants gauge their health expands, so does the possibility that these retrospective reports may be erroneous. For example, Bath [7] found that a global SRH item was more robust in predicting mortality than a self-comparative measure that asked older adults to compare their present health to health five years previously. In a study that compared a global, age-comparative and self-comparative (10 years previous) SRH items it was found that the predictive quality of the self-comparative item became less robust for males after longer follow up periods, whereas none of the SRH items were predictive of mortality in adjusted models for females after 3 and 7.5 year follow-ups [8].

The inconsistent findings regarding the predictive quality of the different SRH items, and the effect that gender may have on this SRH-mortality relationship, warrants further investigation. It is also not clear the extent that these SRH measures predict mortality independently of other potential associated changes, as not all studies have accounted for other known mortality risks, such as demographic variables, health and health behaviours. Furthermore, no study has accounted for the high correlation between the SRH measures by simultaneously modelling the three SRH measures together.

A further consideration is whether the SRH-mortality relationship is constant over time. There is a growing body of evidence that a dynamic evaluation of self-rated health, that is, taking into account the potential time-varying nature of SRH across time, may provide a more authentic depiction of the relationship between subjective health assessments and mortality [9]. Cross-sectional and longitudinal studies suggest SRH does not remain stable across the lifespan [10-13]; therefore using a single occasion measurement of SRH to predict mortality may not take into account the biopsychosocial interactions of health and the lifespan health trajectory [14,15]. The advantage of modelling the relationship between time-varying SRH ratings and mortality is that it enables the modelling of time-varying predictors. Previous studies have found that time-varying measures of Global SRH are a superior predictor of mortality compared to a single fixed measure [15-18]. However, no study to date has investigated how dynamic changes in age-comparative or self-comparative SRH may relate to mortality risk.

The aim of this study is to fill current gaps within the literature regarding the sensitivity of time-varying SRH measures with different reference points to predict mortality in an older adult sample. The unique contributions of the study are (1) the dynamic evaluation of the relationship between *three time-varying *SRH measures (global, age-comparative and self-comparative) on mortality risk, (2) the identification of the *unique *impact of each SRH measure by controlling for time-dependent and time-varying measures of biopsychosocial factors known to increase mortality risk in older adults [15-17], and (3) the comparison of the *independent *and *concurrent *relationship between the three SRH measures on mortality in order to account for the correlation of these measures and further determine their unique predictive quality. While the literature suggests gender differences in the relationship between SRH measures and mortality [e.g. [5,7]], and age-group differences in SRH ratings [e.g. [13,19]], it is not clear if men and women exhibit the same association between SRH and mortality at different ages. Therefore, interactions between SRH items and gender and age-groups are also investigated in order to more fully assess these dimensions of difference.

## Methods

### Sample

The Australian Longitudinal Study of Ageing (ALSA) has been fully described elsewhere [20]. In brief, households with residents over 70 years were identified from the South Australian Electoral Database. The sample was stratified by area, gender, and 5-year cohort groups (70-74, 75-79, 80-84, and ≥ 85) [21]. Males were over-sampled to ensure sufficient numbers in follow up. Of the 2,705 eligible residents 1,477 (55%) agreed to be interviewed. Spouses (>65 years) and co-residents (>70 years) of the sample were also asked to participate, which brought the total number of participants at baseline to 2,087 community dwelling and residential care individuals. The patterns of health care utilization in the final sample were found to be similar to that of the general Australian population [20].

Data collection began in 1992. Baseline and waves 3 (24 months from baseline), 6 (96 months) and 7 (120 months) data consisted of a comprehensive two-hour home interview including questions on demographic, health, medical, psychosocial, and physical status. Waves 2 (12 months from baseline), 4 (36 months), and 5 (60 months) were conducted via short telephone interviews and addressed changes in biopsychosocial factors since last measurement period. Data from all seven waves were included in the current study. Baseline ages ranged from 65 to 103 years of age (mean age = 78.14 years, *SD *= 6.68). In the final wave of data collected the remaining 489 participants were aged between 75 to 102 years (mean age = 84.94 years, *SD *= 4.90). Reasons for non-response at wave 7 were due to death (58.8% of the baseline participants), participants unable to be contacted (2.0%), participants who had moved out of scope of the study (2.3%), and those that refused to be interviewed (13.5%). Table 1 displays the descriptive characteristics for the final sample across all waves.

**Table 1:** Descriptive Statistics for all Waves, and Non-Survivors at Wave 1

	Wave 1		Wave 2	Wave 3	Wave 4	Wave 5	Wave 6	Wave 7
	
	Total	Non-Survivor	Total	Total	Total	Total	Total	Total
	N = 1733	N = 1228	N = 1522	N = 1451	N = 1301	N = 998	N = 740	N = 439
Global SRH (TV)								
- poor	9.3%	12.2%	11.0%	6.7%	10.4%	10.1%	5.3%	6.9%
- fair	22.3%	25.7%	25.0%	25.0%	23.2%	25.5%	22.4%	26.8%
- good	30.0%	30.5%	29.7%	34.3%	31.6%	32.5%	34.7%	41.5%
- very good	29.5%	24.8%	26.2%	26.1%	27.4%	25.0%	27.7%	20.9%
- excellent	8.9%	6.8%	8.1%	7.9%	7.5%	7.0%	9.8%	3.9%
Age-Comp SRH (TV)								
-worse	7.4%	9.1%	7.1%				4.0%	4.3%
- same	32.2%	34.1%	31.3%				36.7%	35.9%
- better	60.4%	56.8%	61.6%			59.3%	59.8%	
Self-Comp SRH (TV)								
-not as good	30.3%	35.4%	36.0%	39.3%	37.5%	43.8%	37.4%	41.6%
- same	56.5%	51.6%	55.4%	50.1%	55.6%	48.0%	52.6%	48.8%
- better	13.1%	12.9%	8.6%	10.6%	6.9%	8.2%	10.%	9.7%
Male	52.6%	58.3%	52.1%	50.1%	49.2%	46.0%	43.8%	38.7%
<$12 000	35.7%	39.5%						
$12 001 - $20 000	45.2%	43.6%						
$20 001 - $30 000	11.4%	9.6%						
$30 001 - $50 000	6.2%	5.7%						
> $50 000	1.4%	1.5%						
≥15 Years Education ^a^	56.0%	41.4%						
Partnered ^b ^(TV)	64.6%	59.9%		48.9%			19.4%	9.8%
Residential Care ^c ^(TV)	6.0%	9.4%		7.8%			14.2%	12.3%
Smoker	8.6%	10.0%						
Ex Smoker	43.3%	44.2%						
Non-Smoker	48.1%	45.8%						
Mean (Standard Deviation)								
Age (TV)	78.7 (6.72)	80.7 (6.37)	79.3 (6.60)	80.0 (6.53)	80.5 (6.40)	81.7 (6.08)	83.6 (5.59)	84.8 (4.77)
Conditions (TV)	2.86 (2.15)	2.94 (2.21)		4.03 (2.53)			4.03 (2.83)	5.18 (3.27)
Medications (TV)	3.26 (2.39)	3.58 (2.43)		3.99 (2.73)			3.89 (2.79)	
ADLs (TV)	0.41 (1.10)	0.52 (1.23)	0.55 (1.43)	1.15 (1.91)	1.10 (1.85)	1.20 (2.08)	1.28 (2.03)	0.84 (1.54)
IADLs (TV)	0.86 (1.58)	1.06 (1.74)	1.33 (2.13)	1.25 (1.45)	0.94 (1.70)	0.90 (1.55)	1.93 (1.84)	0.74 (1.46)
MMSE (TV)	26.90 (4.15)	26.24 (4.60)		27.33 (2.76)			28.06 (2.29)	25.89 (3.71)
Depressive Symptoms (TV)	8.41 (7.49)	9.26 (7.75)		8.36 (7.39)			8.84 (6.89)	5.60 (7.45)

Note: TV = Time-Varying Variables; ADL = Activities of Daily Living; IADL = Instrumental Activities of Daily Living; MMSE = Mini Mental State Examination Score. Reference Category ^a ^- ≤ 14 Years Education; ^b ^- Not Partnered; ^c ^- Community Dwelling.

### Measures

#### Mortality status

Mortality status was established through searches of official death certificates conducted by the Epidemiological Branch of the Department of Health in South Australia and were confirmed by the South Australian Births, Deaths and Marriage bureau [22]. Matching the ALSA respondents to death information was conducted using full name, date of birth, and last known address. During the period from baseline to wave 7 1,228 deaths were recorded (70.9% of sample; 58.3% males). As expected there were significant differences between survivors and non-survivors on predictor variables and covariates. Compared to survivors, non-surviving respondents were more likely to have poorer global (χ^2 ^(4) = 113.69, *p *< .001), age- (χ^2 ^(2) = 51.29, *p *< .001), and self-comparative (χ^2 ^(2) = 25.63, *p *< .001) SRH at baseline. They were also more likely to be male (χ^2 ^(1) = 55.65, *p *< .001), be older at baseline (*t*(1731) = 22.49, *p *< .001), smoke (χ^2 ^(2) = 12.54, *p *= .002), have an income less than $12 000 (χ^2 ^(4) = 32.05, *p *< .001), less than 14 years of education (χ^2 ^(1) = 8.58, *p *= .003), be living in an institution (χ^2 ^(1) = 48.15, *p *< .001), and not have a partner (χ^2 ^(1) = 39.49, *p *< .001). In addition non-survivors were in poorer health at baseline as they were more likely to have more medical conditions (*t*(1731) = 2.65, *p *= .008), be on more medications (*t*(1730) = 8.73, *p *< .001), have greater functional disability (ADL's - *t*(1731) = 6.29, *p *< .001; IADL's - *t*(1731) = 8.27, *p *< .001), poorer cognitive functioning (MMSE; *t*(1702) = -9.36, *p *< .001), and more depressive symptoms (CES-D; *t*(1715) = 6.90, *p *< .001). Table 1 displays the descriptive characteristics for the non-survivors at baseline and the full sample across all waves.

#### Self-rated health (SRH)

Global SRH was measured with the question "How would you rate your overall health at the present time?" (1-'excellent' to 5-'poor'). Age-comparative SRH was measured in response to the question "Would you say your health is better (1), about the same (2) or worse (3) than most people your age?" Self-comparative SRH was worded "Is your health now better (1), about the same (2) or not as good (3) as it was 12 months ago?" Global and self-comparative SRH was measured at all seven waves whilst the age-comparative item was measured at baseline, waves 3, 6, and 7. SRH ratings were reverse coded so that the highest score was equivalent to the most positive health rating.

#### Demographics

Demographic variables included gender, age, community versus residential dwelling, partner status, annual income, and number of years of education. The education question asked participants how old they were when they left school, with possible responses ranging from 1 = never went to school, 2 = under fourteen years, 3 = fourteen years, 4 = fifteen years, to 7 = eighteen or more years. The education variable was dichotomised at the median age category to reflect ≤ 14 years versus ≥ 15 years education [20,22].

#### Physical and functional health and medications

Participants were asked at baseline, wave 3, 6, and 7 if they had been diagnosed and were currently suffering from a heart condition, cancer, or diabetes. In addition participants were shown a prompt card that listed another 38 medical conditions including arthritis, diabetes, and gallstones, and asked to indicate if they suffered from these as well as list any other conditions they had. The total number of conditions currently suffered was summed to create a continuous aggregate score of number of conditions. At baseline, and waves 3 and 6 participants were asked to nominate, and show the interviewer the container, for prescribed and non-prescribed mediations they were currently taking. Number of medications was summed to reflect a continuous variable of total number of medications for each respondent.

Functional status was assessed using the Activities for Daily Living (ADL) and the Instrumental Activities of Daily Living (IADL) measures [23] at all seven waves. The ADL measures difficulties bathing, dressing, eating, using the toilet, and getting around or away from home. IADL questions include ten activities regarding housework, meal preparation, money management and the use of public transport. Scores are coded (0 = "no difficulty" and 1 = "difficulty") and summed so that higher scores indicated greater functional disability.

#### Smoking status

Smoking Status measured current and past smoking of cigarettes, pipe or cigars at baseline. The items were coded to reflect (1) current smoker, (2) ex-smoker and (3) never smoked.

#### Depressive symptoms

Depressive symptoms were measured at baseline and waves 3, 6 and 7, using the Centre for Epidemiology Depression Scale (CES-D), a 20-item questionnaire designed for use in community-based epidemiological studies [24]. A four-point scale was used to assess how an individual felt in the last week, with answers extending from rarely or none of the time (0) to most of the time (3). Summed scores ranged from 0 to 60 with a higher score indicative of more depressive symptoms. The scale had a high level of internal consistency with a Cronbach's alpha coefficient of .85.

#### Cognitive functioning

Cognitive functioning was measured at baseline and waves 3, 6 and 7, with the Mini-Mental State Examinations scale [MMSE: [25]]. The scale assesses orientation to place and time, attention and calculation, and memory recall [20]. The MMSE has been shown to have satisfactory reliability and construct validity and displays a high degree of sensitivity for moderate to severe cognitive impairment [26].

### Statistical analysis

Participants with over 25% observable data missing (n = 354 (16.9%)) were removed from the data set [27] leaving a final sample of 1,733 (52.6% males). This criteria was used as Byrne [27] has shown that model and fit estimates are comparable between a complete data set and one with up to 25% data loss when a full information maximum likelihood imputation method is used. Participants removed from the data set were more likely to be male (χ^2 ^(1) = 15.84, *p *< .001), older at baseline (*t*(2085) = 7.95, *p *< .001), have a greater number of problems with ADL's (*t*(2085) = 3.34, *p *= .001), and IADL's (*t*(2085) = 3.32, *p *= .001), and be taking more medications (*t*(2085) = -4.51, *p *< .001).

Of the final remaining sample 21.3% had < 5% missing data over the seven waves, 14.9% had 6 to 10% missing, 6.8% had 11 to 15% missing, 13.9% had 16 to 20% missing, and 43.1% had >21% missing. As expected, sensitivity analysis revealed that there was a .96 probability (area under the Receiver Operating Characteristic (ROC) curve: *Standard Error *= .005) that a randomly chosen participant in the final sample who had died over the follow up period would have a greater percentage of missing data compared to a randomly chosen survivor. The missing values for the remaining sample were imputed with the maximum likelihood approach of the Expectation Maximization (EM) algorithm method [28]. The EM method uses all available data and alternates the iterative algorithm between estimating missing values from observed responses and parameter estimates and maximises the likelihood for the subsequent full data [29].

Cox regression models were used to analyse the effect of time-varying predictors and covariates on mortality risk (Singer & Willet, 2003). Number of years from baseline interview until death or censorship was the measure for time used in the models. The Cox Regression is a partial likelihood method of estimation which takes into account the number and rank order of deaths in the sample. Because of a 'conditioning argument' [[30]; p.520] within the partial likelihood method no assumptions are made regarding the shape of the baseline hazard function, therefore only the effect of the predictors and covariates are evaluated. The great advantage to using this Cox Regression approach is that a model can be fitted regardless of the baseline hazard function complexity. Singer and Willet [30] describe the probability of the event (mortality) risk is modeled as:

where the time-invariant predictor or covariate in a model is represented by *X*_1*i *_and the time-varying predictor or covariate is represented by *X*_2*ij*. _*h(t*_*ij*_)/*h*_0_(*t*_*j*_) represents an individual's (*i*) mortality hazard ratio (HR) at time *t*_*j *_and is therefore a product of the baseline hazard function *h*_0_, and the individuals true risk score at a given time (i.e. the antilog of each raw coefficient - *β*_1_*X*_1*i *_+ *β*_2 _*X*_2*ij*_). To deal with the unbalanced data (i.e. not all variables are observed at every wave - see Table 1) we carried forward the most recent value of each time-varying predictor to the next wave if it was missing at that wave [30]. Singer and Willet argue that this approach is particularly appropriate to account for the shortfall of predictor information for categorical data when there are complex patterns of temporal variation of observations.

SRH items were treated as ordinal variables with the reference category designated as the most positive rating (i.e. 'excellent' for global; and 'better' for age-and self-comparative). Covariates were computed to reflect time-varying values over the observation periods with the exception of the time-invariant covariates (gender, education, income and smoking status). Income and smoking status were included as categorical variables and therefore baseline measures were used for ease of interpretation. For time-invariant covariates the HR represents the effect of a one-unit change in the related predictor on the raw hazard of mortality over the 10 years of follow-up. For the time-varying predictors and covariates, the HR represents the weighted average of short-term mortality risk across the 10 years follow-up (i.e. the mean of the risks from baseline to first measurement period plus second measurement to third measurement period and so on) [31]. For the categorical predictors the interpretation is essentially the same, except that the HR represents the difference in the risk of mortality compared to the reference group.

The addition of gender and age-group interaction terms into the ordinal models resulted in an additional 32 parameters. To ensure models remained parsimonious [32] SRH items were not identified as categorical in the separate interaction models allowing for an interaction effect to be identified. A significant interaction term resulted in adjusted ordinal models run by group to ascertain group differences.

## Results

### Associations between SRH measures and mortality

Prior to the Cox regression models the relationship between the three SRH measures was investigated. As expected, correlations between the global, age-comparative and self-comparative SRH measures across the seven waves were all significant at *p *< .05. Correlations between global and age-comparative SRH were moderate, ranging from .271 (*p *< .000) at wave 7 to .471 (*p *< .001) at baseline. Similar correlations were found between global and self-comparative SRH, ranging from .278 (*p *< .001) at baseline to .475 (*p *< .001) at wave 4. Correlations were smaller between age-comparative and self-comparative SRH, ranging from .138 at wave 7 to .221 at baseline.

Table 2 shows the unadjusted associations between SRH items and mortality as well as the net effects of SRH items on mortality. 'Poor' global SRH increased the unadjusted risk of mortality by 4.71 times, compared to 'excellent' ratings (model 1). 'Worse' age-comparative SRH increased mortality risk by 2 times compared to 'better' ratings (model 2). 'Not as good' self-comparative ratings increased the mortality risk by 1.23 times compared to 'better' ratings (model 3).

**Table 2:** Hazard Ratios (and 95% Confidence Intervals) Comparing Time-Varying Self-Rated Health (SRH) Models Predicting Mortality (N = 1733)

	**Model 1**	**Model 2**	**Model 3**	**Model 4**	**Model 5**	**Model 6**	**Model 7**
	
	**Global SRH**	**Age-comparative SRH**	**Self-Comparative SRH**	**Global & Age-Comparative**	**Global & Self-Comparative**	**Age-& Self-Comparative**	**All SRH**
	
Global SRH							
Poor^a^	4.71*** (3.48, 6.40)			4.79*** (3.50,6.56)	4.01*** (2.92, 5.52)		4.09*** (2.95, 5.68)
Fair^a^	2.31*** (1.71, 3.11)			2.42*** (1.79, 3.27)	2.06*** (1.52, 2.80)		2.16*** (1.59, 2.95)
Good^a^	1.54*** (1.14, 2.07)			1.59** (1.78, 2.15)	1.46* (1.08, 1.97)		1.51** (1.11, 2.04)
Very Good^a^	1.13 (0.82, 1.54)			1.15 (0.84, 1.57)	1.10 (0.81, 1.52)		1.12 (0.82, 1.54)
Age-Comparative SRH							
Worse ^b^		2.00*** (1.75, 2.52)		1.08 (0.88, 1.31)		1.73*** (1.44, 2.08)	1.07 (0.88, 1.31)
Same ^b^		1.05 (0.93, 1.18)		0.85* (0.75, 0.96)		0.99 (0.88, 1.13)	0.85** (0.75, 0.97)
Self-Comparative SRH							
Not as Good ^c^			1.27* (1.05, 1.54)		0.85 (0.78, 1.16)	1.21* (1.00, 1.47)	0.96 (0.78, 1.17)
Same ^c^			0.67*** (0.56, 0.81)		0.74** (0.61, 0.91)	0.68*** (0.55, 0.82)	0.75** (0.61, 0.91)
							
Null-2LL		16548.63	16548.63	16548.63	16548.63	16548.63	16548.63
-2LL		16264.89	16436.01	16509.75	16485.01	16264.89	16239.88
AIC		16312.89	16474.01	16557.75	16533.01	16312.89	16287.88
BIC		16342.62	16503.74	16587.48	16562.74	16342.62	16317.61

Note: ^a ^- Reference Category is "Excellent"; ^b & c ^"Better". LL = Log Likelihood. ***p < .001; **p < .01; *p < .05.

When global SRH was placed in the same models as age-comparative (model 4) and self-comparative (model 5) SRH the relationship between 'worse' age-comparative and 'not as good' self-comparative ratings and mortality became non-significant. Model 6 revealed that 'worse' age-comparative and 'not as good' self-comparative ratings independently predicted mortality when placed in the same model. In model 7, after accounting for shared variance of all three SRH items, poor global SRH was revealed as the strongest independent predictor of mortality. These results confirm that the global SRH measure accounts for the relationships between the comparative SRH measures and mortality.

Table 3 shows the models adjusted for demographic and health risk factors. In the independent models, after accounting for other mortality risk factors, 'poor', 'fair' or 'good' global SRH ratings and 'worse' age-comparative ratings over time indicate a significant increase in mortality risk for older adults compared to the most positive ratings. In contrast, 'same' self-comparative ratings significantly reduced the mortality risk compared to 'better' over time. In the final full model, all three SRH items are entered to account for overlap between these measures. This model shows that the relationship between the three SRH measures and mortality remains relatively unchanged from the independent models in Table 3. The most notable difference is the reduction in hazard ratio from 3.37 to 2.83 for 'poor' global SRH. This suggests that a poor global rating reflects both age and self comparison processes to some degree.

**Table 3:** Hazard Ratios and 95% Confidence Intervals for Adjusted Self-Rated Health Models Predicting Mortality; the Australian Longitudinal Study of Ageing, 1992 - 2004 (N = 1733)

	Independent SRH Models			Concurrent SRH Model
	**Global SRH**	**Age-Comparative SRH**	**Self-Comparative SRH**	**Full Model**

Global (TV)				
- poor^a^	3.37*** 2.44, 4.65			2.83*** 2.02, 3.98
- fair^a^	1.94*** 1.42, 2.65			1.80*** 1.31, 2.48
- good^a^	1.47* 1.08, 2.00			1.43* 1.05, 1.95
- very good^a^	1.21 0.88, 1.66			1.19 0.87, 1.64
Age-Comp (TV)				
- worse^b^		1.94*** 1.58, 2.38		1.41** 1.14, 1.74
- same^b^		1.13 0.99, 1.29		1.02 0.89, 1.16
Self-Comp (TV)				
- not as good^c^			1.09 0.90, 1.32	0.91 0.74, 1.12
- same^c^			0.72** 0.59, 0.88	0.75** 0.61, 0.93
Male	1.61*** 1.42, 1.84	1.65*** 1.45, 1.87	1.64*** 1.44, 1.87	1.62*** 1.42, 1.84
<$12 000^d^	0.74 0.47, 1.18	0.76 0.48, 1.22	0.73 0.45, 1.16	0.78 0.49, 1.25
$12 001 - $20 000^d^	1.02 0.64, 1.62	1.01 0.64, 1.61	0.97 0.61, 1.55	1.05 0.66, 1.68
$20 001 - $30 000^d^	0.85 0.52, 1.38	0.83 0.51, 1.36	0.80 0.48, 1.30	0.89 0.55, 1.46
$30 001 - $50 000^d^	1.11 0.67, 1.85	1.10 0.66, 1.83	1.04 0.62, 1.73	1.14 0.68, 1.91
≥15 Years Education ^e^	0.93 0.83, 1.05	0.92 0.82, 1.04	0.92 0.82, 1.04	0.93 0.82, 1.04
Partnered (TV) ^f^	0.20*** 0.17, 0.25	0.21*** 0.17, 0.25	0.21*** 0.17, 0.26	0.20*** 0.17, 0.25
Residential Care (TV) ^g^	0.84 0.72, 0.99	0.82* 0.71, 0.96	0.83* 0.71, 0.98	0.86 0.74, 1.00
Age (TV)	1.07*** 1.06, 1.08	1.07*** 1.06, 1.08	1.07*** 1.06, 1.08	1.07*** 1.06, 1.08
Smoker^h^	1.61*** 1.31, 1.98	1.58*** 1.28, 1.94	1.71*** 1.40, 2.11	1.62*** 1.32, 2.00
Ex Smoker^h^	1.13 0.99, 1.29	1.12 0.98, 1.28	1.14 1.00, 1.31	1.12 0.98, 1.28
Number of Conditions (TV)	0.96*** 0.94, 0.98	0.96** 0.94, 0.99	0.96** 0.94, 0.99	0.95*** 0.93, 0.98
Number of Medications (TV)	1.04** 1.01, 1.06	1.05*** 1.03, 1.07	1.05*** 1.02, 1.07	1.04** 1.01, 1.06
ADL (TV)	1.08*** 1.05, 1.11	1.11*** 1.08, 1.14	1.11*** 1.08, 1.14	1.08*** 1.05, 1.11
IADL (TV)	1.01 0.98, 1.04	1.01 0.98, 1.04	1.00 0.98, 1.03	1.00 0.98, 1.03
MMSE (TV)	0.98* 0.97, 1.00	0.99* 0.97, 1.00	0.98** 0.97, 0.99	0.98* 0.97, 1.00
Depressive Symptoms (TV)	1.00 0.99, 1.01	1.00 0.99, 1.01	1.01 1.00, 1.01	1.00 0.99, 1.00
Null-2LL	16548.62	16548.62	16548.62	16548.62
-2LL	15367.35	15452.53	15446.04	15346.16
AIC	15415.35	15500.53	15494.04	15394.16
BIC	15445.08	15530.26	15523.77	15423.89

Note: TV = Time-Varying Variables; ADL = Activities of Daily Living; IADL = Instrumental Activities of Daily Living; MMSE = Mini Mental State Examination Score; LL = log likelihood; AIC = Akaike Information Criterion; BIC = Bayesian Information Criteria. Reference Category ^a ^- "Excellent"; ^b & c ^- "Better"; ^d ^-Income at baseline of $50 000 or more per year;^e^- ≤ 14 Years Education;^f ^- Not Partnered;^g ^- Community Dwelling;^h ^-Non-smoker at baseline. ***p < .001; **p < .01; *p < .05.

### Gender and age by SRH item interactions

As described above separate models investigated gender and age interactions with SRH. The gender by SRH interaction term was not significant (*HR *= 0.99, 95% CI: 0.98, 1.01). However, a significant age by SRH interaction term was found (*HR *= 1.00, 95% CI: 1.00, 1.001). To investigate the interaction effect separate adjusted models for each age-group were conducted (see Table 4). Age-groups were categorised into young-old (65 to 74 years), old-old (75 to 84 years) and oldest-old (85+ years) at baseline, as defined in the gerontological literature [33].

**Table 4:** Hazard Ratios and 95% Confidence Intervals for Concurrent SRH Adjusted Age-Group Models Predicting Mortality; the Australian Longitudinal Study of Ageing, 1992 - 2004

	Young-Old N = 583	Old-Old N = 790	Oldest-Old N = 405
Global (TV)			
- poor^a^	2.98** 1.18, 8.54	3.88*** 2.32, 6.48	1.97* 1.13, 3.44
- fair^a^	2.24 0.95, 5.27	2.30** 1.41, 3.74	1.29 0.77, 2.15
- good^a^	1.90 0.85, 4.26	1.82* 1.14, 2.90	1.01 0.61, 1.67
- very good^a^	1.43 0.62, 3.26	1.40 0.86, 2.28	1.06 0.63, 1.78
Age-Comp (TV)			
- worse^b^	2.57*** 1.60, 4.15	1.17 0.86, 2.28	1.05 0.65, 1.71
- same^b^	1.16 0.83, 1.26	0.91 0.75, 1.10	1.06 0.84, 1.35
Self-Comp (TV)			
- not as good^c^	0.80 0.51, 1.26	1.03 0.77, 1.37	0.76 0.51, 1.21
- same^c^	0.52** 0.32, 0.83	0.89 0.67, 1.19	0.65* 0.44, 0.96
Male	2.52*** 1.85, 3.42	1.54*** 1.28, 1.85	1.48** 1.15, 1.91
<$12 000^d^	1.15 0.56, 5.15	0.77 0.42, 1.39	0.32* 0.13, 0.82
$12 001 - $20 000^d^	1.48 0.34, 6.52	1.19 0.66, 2.16	0.33* 0.13, 0.85
$20 001 - $30 000^d^	1.17 0.26, 5.24	1.13 0.60, 2.13	0.24** 0.09, 0.65
$30 001 - $50 000^d^	1.90 0.40, 9.01	1.41 0.73, 2.72	0.37 0.13, 1.04
≥ 15 Years Education ^e^	0.81 0.60, 1.08	0.98 0.82, 1.16	0.09 0.66, 1.03
Partnered (TV) ^f^	0.15*** 0.10, 0.22	0.17*** 0.13, 0.23	0.34*** 0.21, 0.56
Residential Care (TV) ^g^	1.50 0.81, 2.78	0.89 0.71, 1.12	0.79 0.62, 1.01
Smoker^h^	2.16*** 1.43, 3.25	1.60** 1.18, 2.16	1.08 0.67, 1.74
Ex Smoker^h^	1.23 0.88, 1.72	1.09 0.91, 1.32	0.96 0.75, 1.27
Number of Conditions (TV)	0.95 0.89, 1.00	0.97 0.94, 1.00	0.94** 0.89, 0.98
Number of Medications (TV)	1.04 0.98, 1.11	1.02 0.99, 1.06	1.05* 1.01, 1.09
ADL (TV)	1.06 0.97, 1.16	1.09*** 1.05, 1.14	1.07** 1.02, 1.12
IADL (TV)	1.00 0.92, 1.05	1.00 0.95, 1.04	1.02 0.97, 1.06
MMSE (TV)	1.00 0.96, 1.05	0.98 0.96, 1.01	0.97** 0.95, 0.99
Depressive Symptoms (TV)	0.99 0.97, 1.01	0.99* 0.98, 1.00	1.00 0.99, 1.02
Null-2LL	2550.95	7141.28	3979.88
-2LL	2248.52	6745.06	3858.00
AIC	2598.95	7189.00	4027.88
BIC	2616.49	7210.54	4042.46

Note: TV = Time-Varying Variables; ADL = Activities of Daily Living; IADL = Instrumental Activities of Daily Living; MMSE = Mini Mental State Examination Score; LL = log likelihood; AIC = Akaike Information Criterion; BIC = Bayesian Information Criteria. Reference Category ^a ^- "Excellent"; ^b & c ^- "Better"; ^d ^-Income at baseline of $50 000 or more per year;^e^- ≤ 14 Years Education;^f ^- Not Partnered;^g ^- Community Dwelling;^h ^-Non-smoker at baseline. ***p < .001; **p < .01; *p < .05.

The young-old age-group model revealed that "poor" global and "worse" age-comparative ratings significantly predicted mortality. For the old-old adults age-comparative and self-comparative SRH did not independently predict mortality. Similarly, only 'poor' global ratings were found to significantly predict mortality for the oldest-old age-group. Averaging 'same' self-comparative health resulted in a significant reduction in mortality risk for young-old and oldest-old adults compared to 'better' ratings over time.

## Discussion

Our results indicate that the three SRH items do not have comparable relationships with mortality. These results build on previous findings that SRH measures with different reference points are not interchangeable measures of subjective health for older adults [5,34]. To our knowledge this is the first time the predictive nature of three commonly used SRH items have been compared over time in a dynamic evaluation model. This comparison revealed that, overall, global SRH was the strongest predictor of mortality when taking into account the time-varying nature of the ratings across time, whilst the weakest association was with the self-comparative item.

These findings are contrary to some previous studies that have found that an age-comparative SRH item is a more robust predictor of mortality than a global item for males in a similar age-group [5], or that the three SRH items had similar predictive qualities (55 to 85 year old sample) [8]. However for most previous studies the SRH-mortality associations have been modelled separately for gender [e.g. [5-8]]. The contrasting methodology that was used in the current study revealed non-significant interaction terms for gender in the models, indicating that the relationship between the different SRH measures and mortality was not significantly different for males and females,. Hence separate models for men and women were not justified.

Furthermore, our findings expand the literature because the comparison of the three SRH items was investigated through a dynamic model of SRH and mortality, using time-varying SRH ratings to predict mortality rather than ratings at a single point in time. By modelling the mortality hazard using time-varying predictors and covariates we assessed the cumulative short-term effects of SRH ratings on mortality over a 10 year follow-up period. While well-suited to the global SRH data, the findings indicate that this dynamic evaluation model may not be as suitable for investigating comparative SRH items' association with mortality. In support of this premise additional analysis (not shown here) compared the mortality risk for single measurement SRH ratings (baseline and most recent observation prior to death). These results revealed that the most recent observation of poor self-comparative SRH (rating health as "not as good" as 12 months prior) held a similar association with mortality risk (*HR *= 1.67; *95% CI *= 1.38-2.01) as the most recent 'poor' global SRH rating (*HR *= 1.66; *95% CI *= 1.18-2.34) after adjusting for all SRH measures and other mortality risk factors. In contrast, rating health as worse than others their own age did not significantly predict mortality in the long term (baseline model - *HR *= 1.14; *95% CI *= 0.89-1.46), or on a shorter follow-up period (most-recent observation model - *HR *= 1.03; *95% CI *= 0.83-1.28). These results tentatively suggest that age-comparative SRH is not a good indicator of mortality risk for older adults, whereas considering health to have declined in the past 12 months (self-comparative SRH) may be as good a short term indicator of mortality as poor global SRH.

The difference in predictive quality of these SRH measures is most likely due to the fundamental nature of anchoring the health evaluation to a particular reference point, such as peers or own past health. For example, the age-comparative item may enhance health assessments due to a self-protective 'social downgrading' process [35], whereas the forced temporal aspect of the self-comparative item elicits more negative ratings as it makes recent negative changes in health more salient [36]. Etiologically speaking, self-perceived temporal decline in health could be argued to be a good indicator of imminent mortality risk, as the further analysis above has demonstrated. However, with advanced health decline older adults may perceive that their health cannot get any worse, thus they may begin to rate their health as 'the same' as previous, placing an inherent limitation to the self-comparative item for providing unique mortality information over time. This limitation of the self-comparative SRH measure may also explain our seemingly counterintuitive findings that 'same' self-comparative ratings are protective of mortality risk compared to 'better' ratings. For example, an individual who rates their health as 'better' than the previous year could be reflecting on their experience of recent health issues that may subsequently increase mortality risk. The contrast between the proportion of the current sample who rated their health as "better than others their own age" and "not as good as twelve months ago", along with the small to medium correlations found between the SRH measures, supports the notion that the reference point invokes specific comparison processes which can bias health assessments [14,37], making them less predictive of mortality over time.

Idler and Benyamini [14] and Jylhä [38] argue that the robust SRH-mortality relationship found in global SRH is most likely due to complex, dynamic human judgements that include contextual evaluation frameworks where past and current health is considered along with future health expectations. In the few studies that have compared the determinants of global and comparative SRH items, the global measure has been found to be the most inclusive measure of subjective health in terms of its associations with other factors of health [37,39]. For example, Eriksson et al. [39] found that physical, functional and mental health, health behaviours, and psychosocial factors (such as social support), held significantly stronger associations with global SRH compared to an age-comparative measure. Similarly, the global measure has previously been shown to be the most comprehensive SRH item for the ALSA sample used here [40]. Taken together, these findings suggest that the strong association observed between global SRH and mortality is due to the global measure reflecting an all-encompassing evaluation of health compared to the other SRH items.

In the current study the utility of the SRH items to predict mortality was dependent on age. In particular, the two comparative measures did not provide unique information of mortality risk in adults over 75 years of age. It has been argued that the age-comparative item is not appropriate to use in older populations, or samples with a large age range, due to its sensitivity to age [6,34,38]. The current findings support this notion of age-sensitivity and extend it to the self-comparative item. Further research is needed to clarify whether these comparison effects extend to other age groups or are merely cohort effects.

Whilst the focus of the current study was on the impact of the varying SRH measures on mortality, and the majority of the covariate relationships with mortality are as expected, there are findings in the models that are note worthy. For example, we found a significant protective effect for number of medical conditions. We suggest that the counterintuitive relationship between number of medical conditions and mortality found here are a product of the large number of non-life threatening conditions that were included, such as cataracts, gout, hernia, ingrown toenails, and migraines. The conditions included in the aggregate variable were not weighted here for life-threat, as previous research has suggested that this does not necessarily improve model fit [41], however the combination of stage of disease and comorbidity was found by these authors to increase mortality risk. Together with the current findings it is suggested that future research is needed to ascertain the best way to measure and weight comorbidity in relation to mortality risk.

The major strength of our study is that the large amount of longitudinal data (up to seven waves spanning 12 years) allowed for comprehensive health models to be tested in a time-varying approach. Furthermore, the mortality relationship of each SRH item was established by directly comparing the items whilst accounting for shared variance with other SRH items and health covariates. A limitation of the data set is the unbalanced data collection (i.e. not all measures were observed at each wave) and the different modes of data collection (face-to-face versus telephone interview). Whilst, previous research has supported the reliability of telephone interviewing and the strong correlation between this mode with face-to-face interviewing in established samples [e.g. [42,43]], the unbalanced data may require care in the interpretation of results. Singer and Willet [30] argue that the method of imputing the time-varying predictors when they are not observed, by carrying forward the most recent value (as was used here, see Statistical Analysis section), is most likely to result in a conservative estimate. It should also be noted that the small number of oldest-old adults at baseline remaining in the wave 6 and 7 samples could limit our findings, as small sample sizes may result in reduction of statistical power to detect significant effects [29]. Baseline selection effects of this age group must also be taken into account [44], along with possible sample attrition due to causes other than mortality. For example, the significant differences in characteristics of the excluded participants (due to missing data, see Statistical Analysis section above) suggest a possible bias as the oldest-old, males, and those with increased functional difficulties and number of medications were less likely to be included in the final sample. However, the finding that 'poor' global SRH predicted mortality for the oldest-old age group in the adjusted models, as did being male and having increased ADL's, suggests our results are more likely to be an underestimation of effects. Therefore the relationship between time-varying SRH and mortality risk may in fact be stronger than is indicated here.

## Conclusions

In conclusion, global, age-comparative and self-comparative SRH items embody unique, age-sensitive, associations with mortality over time. Researchers should exercise caution when pooling or harmonising SRH items as they are not comparable measures of health. Future research investigating the potential for time-varying, and even change in, SRH measures to predict other major health outcomes, such as functional disability and health care utilisation, may extend the application of SRH items for indicators of population health.

The age sensitivity of the comparative SRH measures suggests they should be used with caution in older adult populations, particularly if used for predicting mortality. Furthermore, the usefulness of tracking age- and self-comparative measures to predict mortality is limited by the anchoring of the evaluation to the reference point. In contrast, 'poor' global is a robust predictor of mortality across age groups over time, indicating that this is the most reliable measure of self-perceived health for older adults.

## Competing interests

This study was funded by the South Australian Health Commission, the Australian Rotary Health Research Fund, the US National Institute of Health (Grant No. AG 08523-02) and the National Health and Medical Research Council (NHMRC; Grant No.229936). KJA is supported by NHMRC Fellowship No.366756.

## Authors' contributions

Author KSC designed the current study, conducted the literature review, data analyses and interpretation of the data, and drafted the article. Author's KJA and MAL contributed to the draft and supervised the study's analytical strategy. All authors have read and approved the final manuscript.

## Pre-publication history

The pre-publication history for this paper can be accessed here:

http://www.biomedcentral.com/1471-2318/10/18/prepub
